# *Helicobacter pylori* Infection Induces Anemia, Depletes Serum Iron Storage, and Alters Local Iron-Related and Adult Brain Gene Expression in Male INS-GAS Mice

**DOI:** 10.1371/journal.pone.0142630

**Published:** 2015-11-17

**Authors:** Monika Burns, Sureshkumar Muthupalani, Zhongming Ge, Timothy C. Wang, Vasudevan Bakthavatchalu, Catriona Cunningham, Kathleen Ennis, Michael Georgieff, James G. Fox

**Affiliations:** 1 Division of Comparative Medicine, Massachusetts Institute of Technology, Cambridge, Massachusetts, United States of America; 2 Department of Biological Engineering, Massachusetts Institute of Technology, Cambridge, Massachusetts, United States of America; 3 Department of Medicine, Columbia University, New York, New York, United States of America; 4 School of Medicine and Dentistry, University of Aberdeen, Aberdeen, Scotland, United Kingdom; 5 Division of Neonatology, Department of Pediatrics, University of Minnesota Medical School, Minneapolis, Minnesota, United States of America; Institut Pasteur Paris, FRANCE

## Abstract

Iron deficiency anemia (IDA) affects > 500 million people worldwide, and is linked to impaired cognitive development and function in children. *Helicobacter pylori*, a class 1 carcinogen, infects about half of the world’s population, thus creating a high likelihood of overlapping risk. This study determined the effect of *H*. *pylori* infection on iron homeostasis in INS-GAS mice. Two replicates of INS-GAS/FVB male mice (n = 9-12/group) were dosed with *H*. *pylori* (*Hp*) strain SS1 or sham dosed at 6–9 weeks of age, and were necropsied at 27–29 weeks of age. Hematologic and serum iron parameters were evaluated, as was gene expression in gastric and brain tissues. Serum ferritin was lower in *Hp* SS1-infected mice than uninfected mice (p < 0.0001). Infected mice had a lower red blood cell count (p<0.0001), hematocrit (p < 0.001), and hemoglobin concentration (p <0.0001) than uninfected mice. Relative expression of gastric hepcidin antimicrobial peptide (*Hamp*) was downregulated in mice infected with *Hp* SS1 compared to sham-dosed controls (p<0.001). Expression of bone morphogenic protein 4 (*Bmp4)*, a growth factor upstream of hepcidin, was downregulated in gastric tissue of *Hp* SS1-infected mice (p<0.001). *Hp* SS1-infected mice had downregulated brain expression of tyrosine hydroxylase (*Th)* (p = 0.02). Expression of iron-responsive genes involved in myelination (myelin basic protein (*Mbp)* and proteolipid protein 2 (*Plp2*)) was downregulated in infected mice (p = 0.001 and p = 0.02). Expression of synaptic plasticity markers (brain derived neurotrophic factor 3 (*Bdnf3)*, *Psd95* (a membrane associated guanylate kinase), and insulin-like growth factor 1 (*Igf1*)) was also downregulated in *Hp* SS1-infected mice (p = 0.09, p = 0.04, p = 0.02 respectively). Infection of male INS-GAS mice with *Hp* SS1, without concurrent dietary iron deficiency, depleted serum ferritin, deregulated gastric and hepatic expression of iron regulatory genes, and altered iron-dependent neural processes. The use of *Hp* SS1-infected INS-GAS mice will be an appropriate animal model for further study of the effects of concurrent *H*. *pylori* infection and anemia on iron homeostasis and adult iron-dependent brain gene expression.

## Introduction

Despite eradication efforts within defined groups and its decline due to improved socio-economic conditions over the past 20 years, *Helicobacter pylori* infection remains prevalent worldwide, with infection rates ranging from approximately 20 to 80% in developed and developing countries respectively [1–3]. Infection with *H*. *pylori* results in gastric inflammation, and may lead to clinical outcomes including peptic ulcers, atrophic gastritis, and gastric cancer [4–6]. *H*. *pylori* is categorized as a Class 1 carcinogen, and is estimated to be responsible for 60% of gastric cancer cases worldwide [7].

Over time, a multitude of extragastric effects of *H*. *pylori* infection have been hypothesized, many of which have not been completely defined [8]. These extragastric disorders may be directly caused by or related to *H*. *pylori* infection, and include endocrine disorders, ischemic diseases, and neurological disorders, among others [8]. Multiple meta-analyses have provided convincing evidence that a causal association exists between *H*. *pylori* infection and iron deficiency anemia (IDA) [9, 10]. Eradication of *H*. *pylori* improves iron status and anemia in human patients with IDA [10]. Since the populations most likely to be affected by *H*. *pylori* and ID overlap throughout much of the developing world, there is a need to better understand the potential clinical sequelae that could result from *H*. *pylori* infection and ID co-morbidity.

Iron deficiency (ID) is the most common nutritional deficiency in the world. ID is an important cause of anemia (IDA), which is categorized by WHO as a critical public health issue [11]. Over 2 billion people are at risk of being affected by ID worldwide, and while it remains an issue in the developed world, it is especially problematic in developing countries [12]. Iron is essential to physiological homeostasis in many capacities; one of its most important functions is to transport oxygen throughout the body as a component of hemoglobin in red blood cells. It is also crucial for normal brain development in children [13]. Iron plays a role as a cofactor in many redox reactions within the body as well, including those related to energy production, intermediate metabolism, neurotransmitter maintenance, and the generation of reactive oxygen species [14]. The usable pool of iron is limited, and iron found in the diets of humans and animals tends to be poorly bioavailable [15]. Iron deficiency, whether caused by a nutritional deficiency or through chronic blood loss due to endoparasitism or neoplasia, is a serious public health problem worldwide [13].

Infectious diseases such as *H*. *pylori* have gained recognition as important contributors to the development of ID/IDA, and there is interest in elucidating the mechanism of pathogenesis. *H*. *pylori* is able to disturb host cell polarity to use the apical cell surface as a replicative niche, and disrupts host intracellular iron-trafficking *in vitro*. This is a novel mechanism of iron acquisition by *H*. *pylori*, and represents a means by which *H*. *pylori* infection results in decreased host ability to transport iron for further use by the body [16]. The pathogen is competing with the host for iron, thereby decreasing the amount of iron available for use by the host. *H*. *pylori* infection increases the amount of iron in AGS cells, perturbing intracellular iron homeostasis [17]. *In vivo* studies have also shown that gastric *Helicobacter* infection alters iron status in mouse and gerbil models [18–21]. Previous studies have demonstrated increased virulence in *H*. *pylori* isolates from iron-depleted gerbils when compared to isolates from iron-replete gerbils; the number of pili per bacterial cell was significantly higher in *H*. *pylori* isolates recovered from iron-depleted gerbils 12 weeks after infection [18]. The authors documented an increase in severity and accelerated development of gastric injury and carcinoma in the same *H*. *pylori*-infected, iron-depleted gerbils. Another mechanism by which *H*. *pylori* infection may contribute to iron deficiency is through alteration of gastric pH. *H*. *pylori* infection may progress to atrophic gastritis with loss of parietal cells, which results in hypochlorhydria or achlorhydria, potentially causing or exacerbating IDA [22]. This occurs because the absorption of dietary iron is dependent upon an acidic gastric environment for reduction of ferric iron to the ferrous form; this is the only form of iron that can be transported across the luminal membrane of enterocytes. Gastrin levels, at both the serum and local level, also affect absorption of iron in the stomach in both mice and humans [23].

There is a growing body of literature that supports the important role that the gut-brain axis plays in central nervous system function [24]. As a gastric pathogen, *H*. *pylori* may influence the gut-brain axis in infected individuals, though this has not been proven in humans or animal models [25, 26]. There may be a link between ID, altered microbiome, and attention deficit hyperactivity disorder (ADHD) [27]. The precise mechanisms and developmental timing of how ID affects the developing brain has been a topic of considerable interest for decades, but remains incompletely defined. There are numerous neurological and cognitive consequences of early life ID, in humans and animals, including motor skill deficits, developmental delays, cognitive impairment, depression, anxiety, and behavioral disorders [28]. Children who experience ID in infancy score lower on tests of mental and motor functioning for >10 years after iron supplementation [29, 30]. Adequate brain iron is crucial for neurotransmitter metabolism, myelin formation, and brain energy metabolism [31]. As these processes are all essential to proper brain function, their functional dysregulation in ID likely contributes to the cognitive deficits that have been documented in patients with ID. The severity of neurological sequelae seen as a result of ID increases the importance of dissecting the role of *H*. *pylori* in ID pathogenesis.

Although evidence of an increased disease burden exists in patients with concurrent *H*. *pylori* and ID/IDA, further studies are needed to define pathophysiology, neurological consequences, and at-risk populations. While previous studies have documented the negative effect of chronic *Helicobacter felis* infection on iron homeostasis in transgenic INS-GAS mice, the impact on the hematologic parameters used to identify and characterize anemia has not been described [19]. The effects of *H*. *pylori* infection and concurrent iron deficiency on development of anemia have also not been previously explored in a mouse model. The INS-GAS mouse model has been used by our laboratory as well as others as a mouse model of human gastric adenocarcinoma [32–34]. One of the primary goals of this study was to define the effects of chronic *H*. *pylori* infection on hematologic parameters and systemic iron homeostasis of male INS-GAS mice. We hypothesized that infection of male INS-GAS mice with *H*. *pylori* would result in significantly decreased red blood cell indices and lower serum iron storage levels than found in sham-dosed controls. Another primary aim was to examine the impact of long-term *H*. *pylori* infection on iron-related gene expression in gastric and liver tissue, and to evaluate the effect of infection on iron-related brain gene expression; particularly expression of genes related to dopamine metabolism, myelination, and synaptic plasticity.

## Materials and Methods

### Animals

The Massachusetts Institute of Technology (MIT) Committee on Animal Care approved the use of mice in this study (Animal Welfare Assurance #A3125-01). Transgenic male INS-GAS mice on a FVB/N background, bred and maintained at MIT, were used for this study. Male mice were used, as development of gastric cancer after infection with *H*. *pylori* has previously been shown to be a gender specific effect [32]. This study was performed in two identical replicates (Study 1 and Study 2). In Study 1, 12 mice were dosed with *H*. *pylori* SS1 (see below), and 9 mice were sham-dosed as controls. In Study 2, 9 mice were dosed with *H*. *pylori* SS1, and 9 mice were sham-dosed as controls.

The results described in this study reflect results from both studies combined, except where otherwise noted. The gastric phenotype of INS-GAS mice, which overexpress human gastrin due to the presence of the insulin promoter upstream of human gastrin coding sequences, was first characterized in 2000, and the mice have since been used extensively as a robust model to study *H*. *pylori* infection [35, 36]. All animals were housed in facilities accredited by the Association for Assessment and Accreditation of Laboratory Animal Care (AAALAC) International. Temperature was monitored and recorded at 20±1°C and relative humidity of 30–70%. All mice were maintained as specific pathogen-free (SPF) of murine viruses, bacteria (including *Helicobacter spp*.), and parasites. Microisolator caging was used for group housing of mice, and mice were maintained on hardwood chip bedding (SaniChip).

### Diet

Mice were allowed *ad libitum* access to an iron-replete (380 ppm iron) standard rodent chow throughout the entire duration of the study (ProLab RMH 3000, Purina Mills, St. Louis, MO).

### Experimental infection with *Helicobacter pylori*

*H*. *pylori* SS1 was used in this study. *H*. *pylori* SS1 (Sydney strain 1) is the most commonly used and best characterized strain of *H*. *pylori* for use in mouse models. It does not contain a functional *cag* pathogenicity island (cag-PAI) [33, 36]. All *H*. *pylori* isolates were maintained and cultured in-house using previously established culture methods [33]. INS-GAS mice were orally gavaged with 1X10^8^ colony forming units of *H*. *pylori* SS1 in 200 μl of sterile freeze media every other day for a total of 3 doses. Mice were either dosed with *H*. *pylori* SS1 or sham-dosed with 200 μl sterile freeze media between 6 and 9 weeks of age. Infection status of the mice at the end of the experiment was evaluated via quantitative PCR (qPCR) of gastric tissue. Mice were euthanized at 26–28 weeks post infection via carbon dioxide overdose.

### Complete Blood Count

Whole blood samples (1 mL) were collected at necropsy in anesthetized mice via cardiac puncture, and immediately placed into 1 mL EDTA-coated anticoagulant blood collection tubes. Samples were processed within 4 hours of collection at the Massachusetts General Hospital Center for Comparative Medicine Clinical Pathology lab (Boston, MA). Complete Blood Count (CBC) analysis was performed on a HESKA HemaTrue Veterinary Analyzer (Loveland, CO), using an impedance-based method for counting white blood cells, red blood cells, and platelets. Hemoglobin was measured on the HemaTrue Analyzer using a cyanide-free method and measured by a spectrophotometer set to 535 nm.

### Serum Iron Parameters

Whole blood samples (1 mL) were collected at necropsy under CO_2_ via cardiac puncture, and immediately placed into 1 mL blood collection tubes containing no anticoagulant. Tubes were centrifuged after coagulation, and serum was collected and separated into individual tubes. Serum iron levels and unsaturated iron binding capacity (UIBC) were evaluated using an Architect c8200 Chemistry Analyzer (Abbott Diagnostics, Illinois). (Total iron binding capacity (TIBC) and transferrin saturation (Tfr %) were calculated using serum iron and UIBC values using the following equations: TIBC = UIBC + Serum Iron; Tfr% = Serum Iron/TIBC.

### Liver Iron Concentration

Liver iron was assessed using an Iron Assay Kit from Sigma Aldrich (St. Louis, MO). From each liver sample (flash-frozen in liquid nitrogen at necropsy, kept at -80C), 10 mg of tissue was prepared according to instructions, and then compared to iron standards with a colorimetric detection process described in the manufacturer’s instructions.

### Serum Ferritin

Serum ferritin concentrations were measured using a mouse specific enzyme-linked immunosorbent assay (ELISA) kit (Kamiya Biomedical, Seattle, WA) according to the instructions provided by the manufacturer. Serum ferritin was evaluated as a more reliable adjunct indicator to serum iron, as transferrin-bound iron is highly variable [37, 38].

### Necropsy

Whole brain was collected immediately post-mortem, submerged in liquid nitrogen, and stored at -80°C. RNA extraction and evaluation of mRNA expression was performed via quantitative PCR (qPCR). Stomach and proximal duodenum was sectioned beginning at the greater curvature, and homogenous linear sections were collected for histopathology, DNA and RNA extraction to evaluate mRNA expression and *H*. *pylori* colonization level. Stomach tissue samples (linear strips from the squamo-columnar junction through proximal duodenum) collected for DNA and RNA extraction were flash-frozen in liquid nitrogen and stored at -80°C. Strips containing proximal duodenum were submitted for histopathologic analysis. Sterile razor blades were used for stomach sectioning. Sections of stomach, liver, spleen, and femur were collected, trimmed, and fixed overnight in 10% neutral buffered formalin. All tissues were processed, embedded in paraffin, cut into 4-μm-thick sections, and stained with hematoxylin and eosin. Additional liver and spleen sections were stained with Prussian Blue for assessment of iron content. Spleens from Study 2 were weighed, and the percentage of spleen mass in relation to total body mass was calculated.

### Histopathologic evaluation and lesion scoring

All tissues were evaluated by a board-certified comparative pathologist (S.M) blinded to both treatment groups and sample identity. Gastric lesions were scored on an ascending scale from 0 to 4 using previously established criteria [39–41]. Lesions related to inflammation, epithelial defects, oxyntic atrophy, epithelial hyperplasia, pseudo-pyloric metaplasia, dysplasia, hyalinosis, and mucus metaplasia were scored. A gastric histopathologic assessment index (GHAI) score was calculated for each animal via the summation of all individual category scores.

### Bone Marrow Cytology

At the time of necropsy, femurs were isolated and removed from surrounding tissue. Femurs were transected longitudinally using a sterile razor blade, and brush preps were prepared using a small paintbrush coated with phosphate buffered saline. Bone marrow was collected on the brush, and applied in a thin film to glass slides. Slides were allowed to dry prior to staining. Cytological evaluation was performed by a board-certified veterinary pathologist.

### Real Time Quantitative PCR

Relative mRNA expression for specific genes was assessed in both gastric and liver tissue. Stomachs were incised along the greater curvature, opened, and sectioned into three linear strips. One strip, containing corpus, body, and antrum, was used for RT-qPCR. Relative expression of hepcidin antimicrobial peptide (*Hamp*), bone morphogenic peptide 4(*Bmp4)*, lipocalin 2 *(Lcn2*), interferon gamma (*IFNγ*), tumor necrosis factor alpha (*TNFα)*, interleukin one beta (*IL1β*), and interleukin ten (IL10) was evaluated. Total RNA from liver and stomach was prepared using Trizol reagent (Life Technologies) and RNeasy Kit (Qiagen) respectively, according to manufacturer instructions. Two μg of total RNA from each sample was converted to cDNA using a High-Capacity cDNA Reverse Transcriptase Kit (Applied Biosciences). Levels of *Bmp4*, *Hamp*, *Lcn2*, *IFNγ*, *TNFα*, *IL1β*, and *IL10* were measured by quantitative PCR using commercially available primer/probes (TaqMan Gene Expression Assays) in the 7500 Fast Sequence Detection System. C_T_ values were normalized to an endogenous control glyceraldehyde-3-phosphate dehydrogenase mRNA, and were expressed as relative fold-change compared to sham-dosed animals using the comparative C_T_ method.

In addition to gastric and liver tissue, whole brain mRNA expression was assessed from Study 2 mice. Total RNA from whole brain tissue was extracted (RNAqueous kit, Lifetechnologies; Carlsbad, CA) and first strand cDNA was generated using 2 μg of RNA (high capacity RNA-to-cDNA kit, Lifetechnologies; Carlsbad, CA). Relative mRNA expression of transferrin receptor (*TfR*), divalent metal transporter (*Slc11a2*), tumor necrosis factor alpha (*Tnfα*), interleukin 6 (*IL-6*), tyrosine hydroxylase (*Th*), myelin basic protein (*Mbp*), proteolipid protein 2 (*Plp2*), brain derived neurotrophic factor 3 (*Bdnf3*), a membrane-associated guanylate kinase (*Psd95*), and insulin-like growth factor 1 (*Igf1*) were measured using quantitative PCR. Commercially available primer/probes (Lifetechnologies; Carlsbad, CA) and Taqman master-mix (Roche Life Science; Indianapolis, IN) were used on a Stratagene MX300P qPCR system (Agilent Technologies; Santa Clara, CA). Samples were assayed in duplicate and normalized against ribosomal protein S18.

### Western Blot

Protein concentrations of BDNF, MBP, & PSD95 were determined using previously published methods with minor modifications (n = 4–5 per group) [42]. Briefly, 20μg of whole brain homogenate was separated on an SDS page gel (Thermo Fisher Scientific, Waltham, MA) and transferred onto PVD membrane. Membranes were incubated for 1 hr in blocking buffer (Rockland Immunochemicals, Limerick, PA) and then incubated overnight at 4°C with mouse anti-ß-actin antibody (1:5000; Abcam, Cambridge, MA) in addition with one of the following rabbit antibodies against BDNF or MBP, or anti-goat PSD95 (1:1000; Abcam, Cambridge, MA). Following primary antibody incubation, membranes were rinsed and incubated at room temperature for 1 hour with appropriate fluorescent secondary antibodies. The membranes were imaged (Odyssey Infrared Imaging System; LI-COR Biosciences, Lincoln, NE) and the intensity of the target protein relative to β-actin was determined.

### Colonization levels of *H*. *pylori* SS1

DNA was extracted from stomach tissue using a High Pure PCR Template Preparation Kit (Roche Diagnostics, Indianapolis, IN). Colonization levels of *H*. *pylori* SS1 within the gastric mucosa were quantified using *H*. *pylori* DNA-specific primer/probes in the 7500 Fast Sequence Detection System (Applied Biosystems) using a previously developed gene target based on the nucleotide sequence of the *H*. *pylori* ureB gene [43]. To quantify mouse DNA, samples were probed with appropriate18S rRNA gene-based primers as previously published [44]. Copy numbers of *H*. *pylori* were defined per μg of mouse DNA.

### Statistical Analysis

All statistical analyses were performed using GraphPad Prism Version 6.01. Sample distributions and variances were evaluated, and statistical analyses were performed using parametric or non-parametric methods based on normality and equivalence of variance. For parametric evaluation, student’s t-tests were used, and Mann-Whitney tests were used as a non-parametric method of comparing group means. Statistical significance was designated as a p-value <0.05.

## Results

### Chronic *H*. *pylori* infection depleted serum iron stores

**Fig 1:**
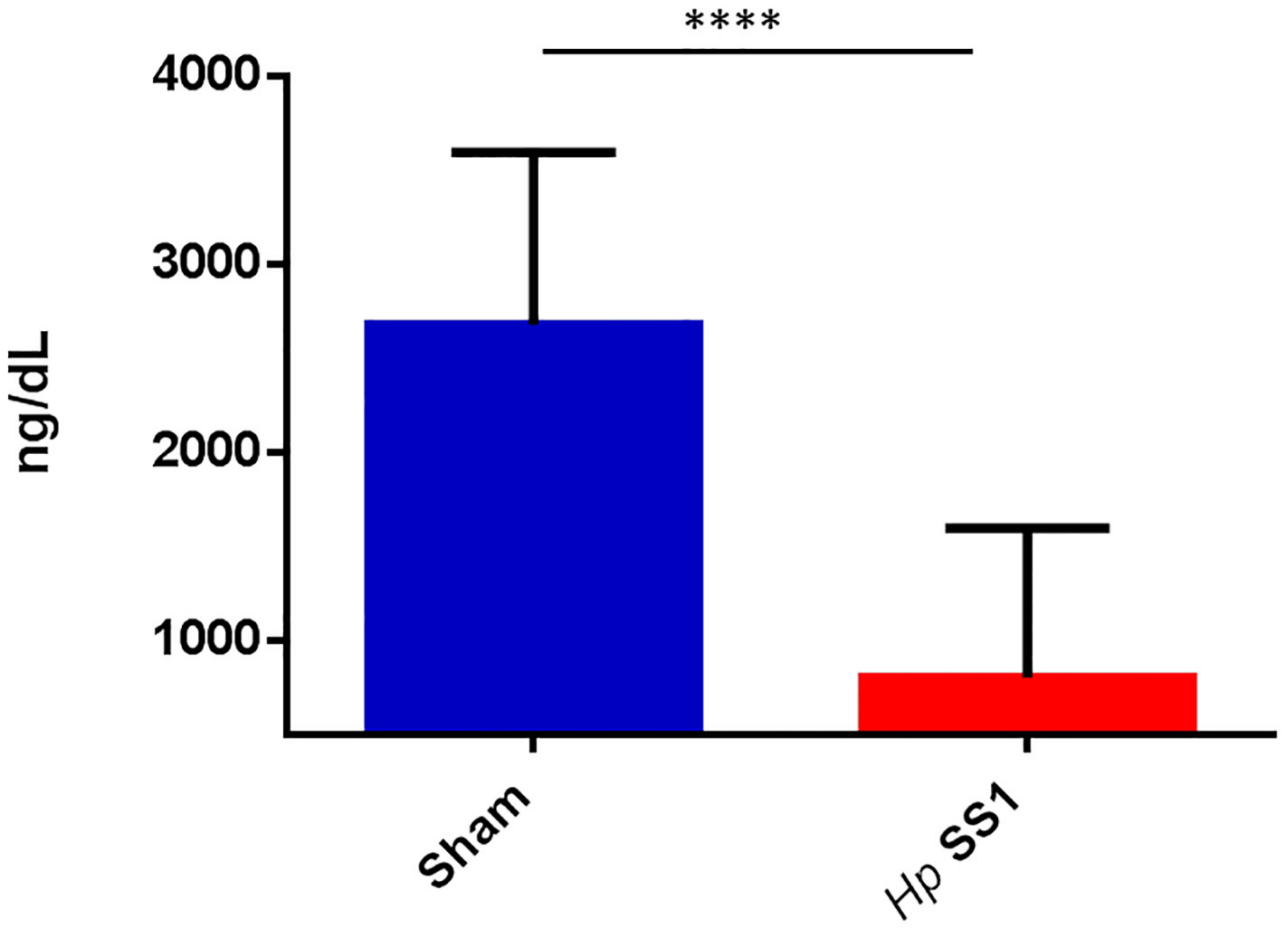
INS-GAS/FVB mice chronically infected with *H*. *pylori* have lower serum ferritin than uninfected mice. Serum ferritin concentration was significantly lower in mice infected with *H*. *pylori* SS1 when compared to sham-dosed controls. (****) = p<0.0001.

Ferritin, an intracellular iron-binding protein that is an important component of the body iron storage pool, was decreased in *H*. *pylori*-infected INS-GAS mice. Serum ferritin concentration was significantly lower in mice infected with *H*. *pylori* SS1 when compared to sham-dosed control mice (Fig 1) (p<0.0001). *H*. *pylori* infection status did not affect serum concentrations of iron bound to transferrin or unsaturated iron binding capacity (data not shown).

There were no differences in liver iron concentration detected between *H*. *pylori*-infected mice and sham-dosed controls. Histopathologic evaluation of paraffin embedded tissues stained with Prussian Blue stain confirmed that there was no difference in iron concentration between *H*. *pylori*-infected and uninfected mice (data not shown).

### *H*. *pylori* infection altered mRNA expression profile of gastric inflammatory cytokines, and gastric and hepatic iron-related genes

**Fig 2:**
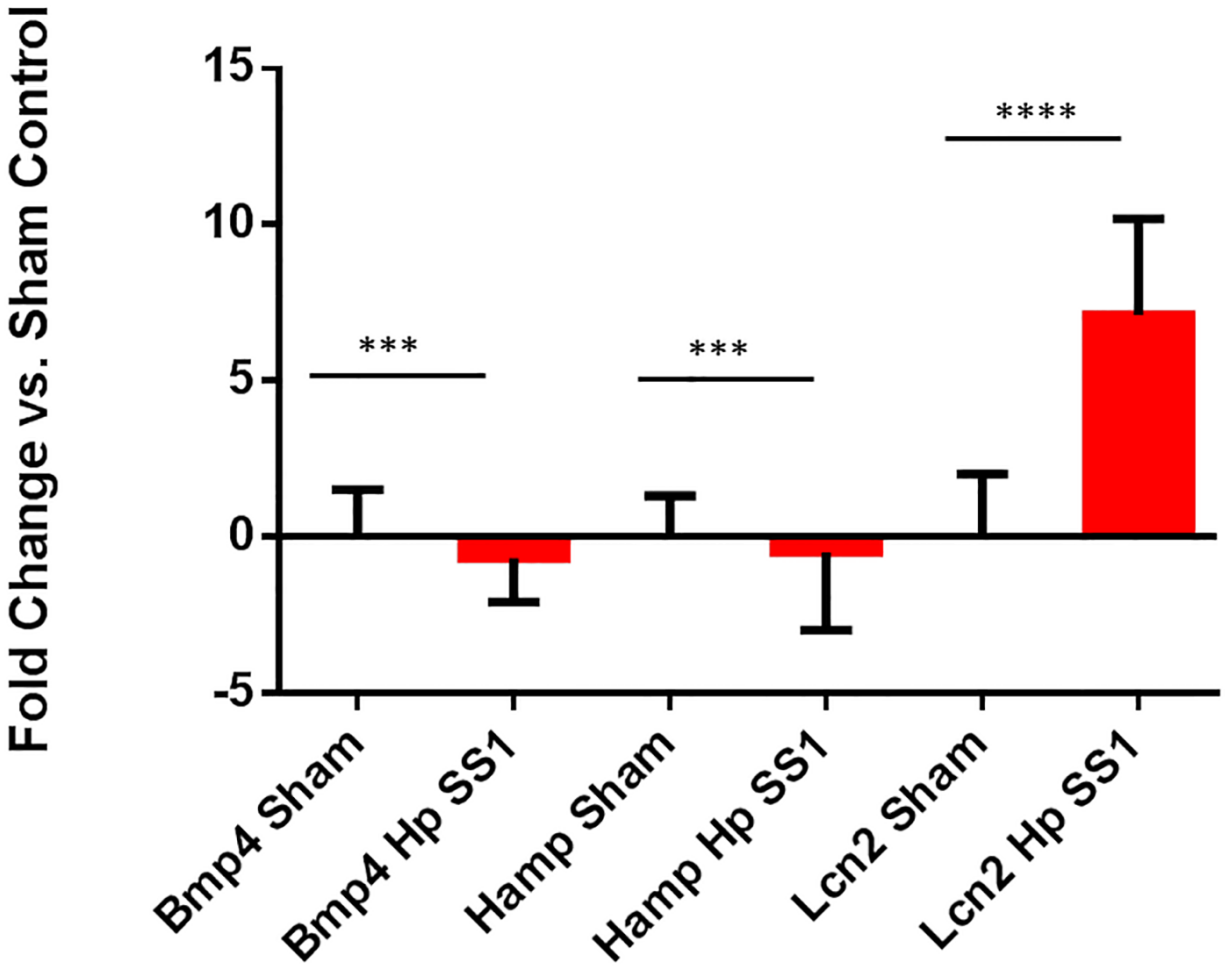
Expression of gastric iron regulatory genes was downregulated in INS-GAS mice chronically infected with *H*. *pylori*. Gastric tissue expression of bone morphogenic protein 4 (*Bmp4)* and hepcidin antimicrobial peptide (*Hamp)* mRNA was significantly downregulated, while Lipocalin 2 (*Lcn2)* expression was upregulated, in mice infected with *H*. *pylori* SS1. (***) = p<0.001; (****) = p<0.0001.

**Fig 3:**
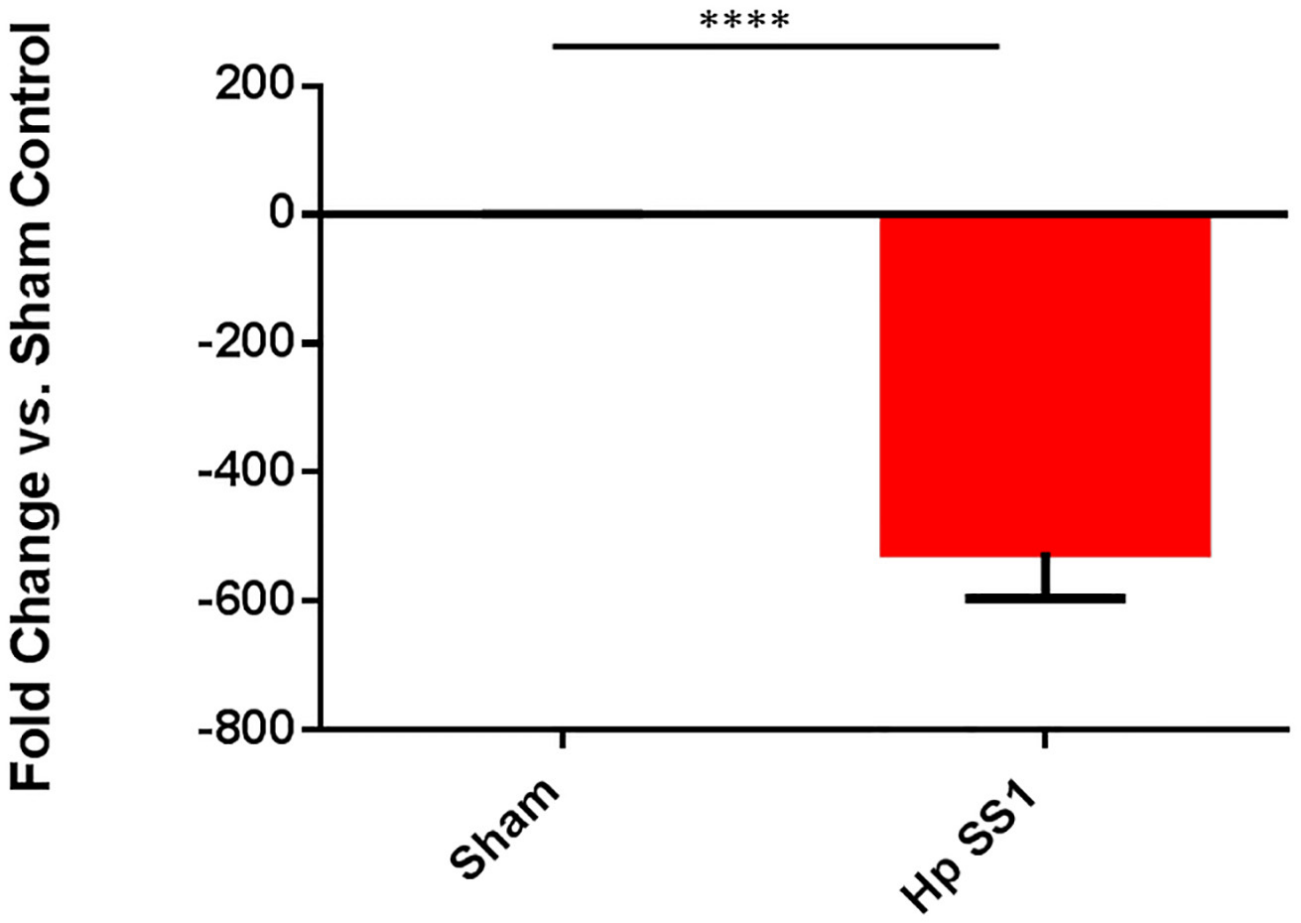
Expression of hepcidin is downregulated in hepatic tissue isolated from *H*. *pylori* -infected mice. Expression of hepcidin antimicrobial peptide (*Hamp)* was lower in *H*. *pylori—*infected mice. (****) = p<0.0001.

Expression of gastric iron regulatory genes was downregulated in INS-GAS mice chronically colonized with *H*. *pylori*. Hepcidin, a peptide hormone produced primarily by hepatic tissue, but also by gastric tissue, controls both uptake of iron into small intestinal epithelial cells and export of iron into circulation via regulation of ferroportin 1 expression. Relative expression levels of hepcidin antimicrobial peptide (*Hamp*) and bone morphogenic protein 4 (*Bmp4*), a growth factor upstream of hepcidin, were evaluated in mouse gastric tissue. Expression of *Bmp4* mRNA was significantly downregulated in mice infected with *H*. *pylori* SS1 when compared to sham-dosed controls (Fig 2) (p<0.001). *H*. *pylori* infection also significantly downregulated expression of gastric *Hamp* in *H*. *pylori*-infected mice when compared to sham-dosed control mice (Fig 2)(p<0.001),). *Lcn2* expression was evaluated as a marker of inflammation, and was upregulated in mice infected with *H*. *pylori* SS1 (Fig 2)(p<0.0001). Hepatic *Hamp* expression was also significantly downregulated in mice infected with *H*. *pylori* SS1 when compared to both sham-dosed control mice (Fig 3) (p<0.0001).

**Fig 4:**
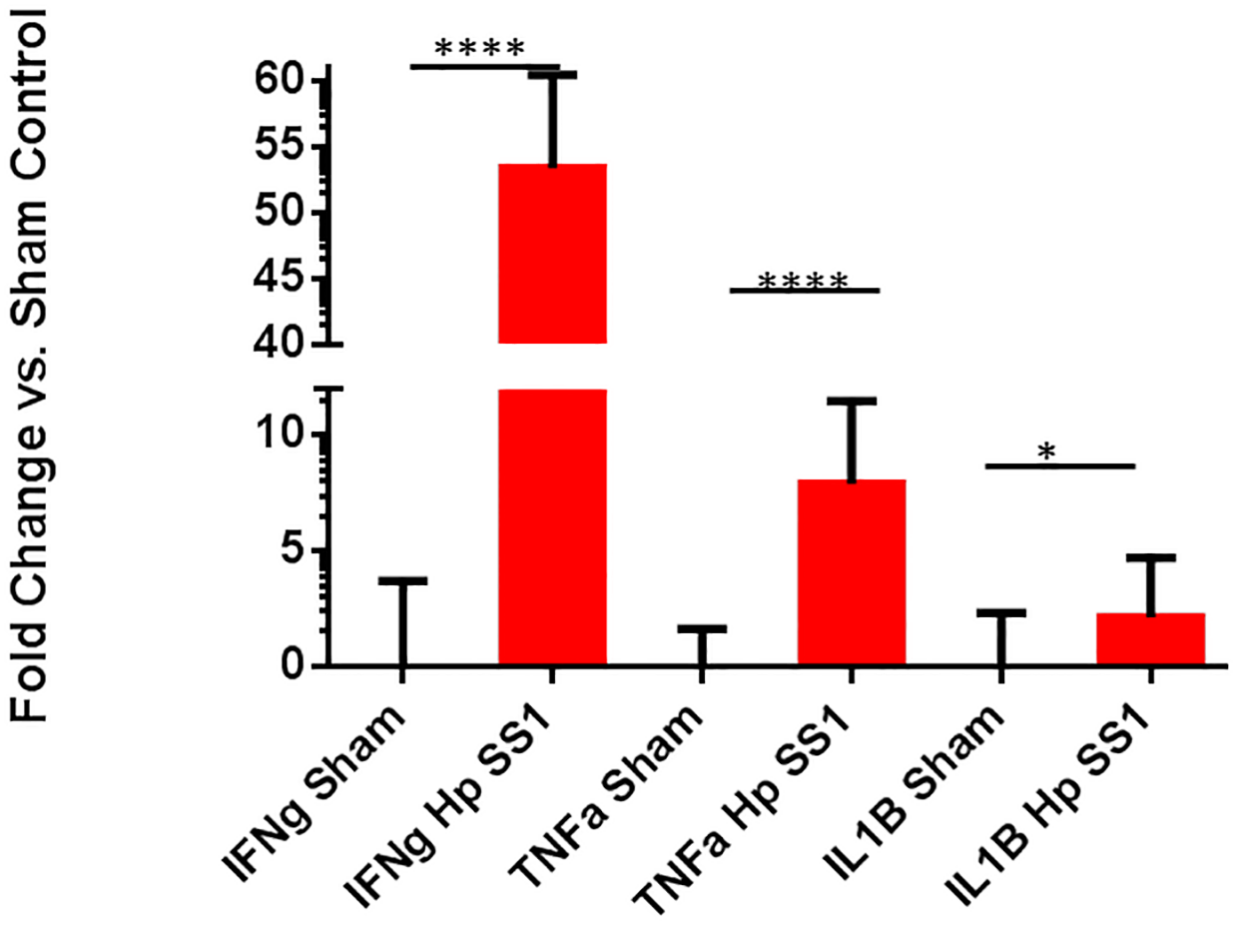
Infection with *H*. *pylori* upregulated gastric inflammatory cytokine expression. Expression of interferon gamma (IFNγ), tumor necrosis factor alpha (TNFα), and interleukin one beta (IL1β) was significantly upregulated in *H*. *pylori*-infected mice compared to uninfected mice. (*) = p<0.05; (****) = p<0.0001.

Infection with *H*. *pylori* significantly upregulated gastric expression of pro-inflammatory cytokines including: interferon gamma (IFNγ) (Fig 4)(p<0.0001)., tumor necrosis factor alpha (TNFα), (Fig 4)(p<0.0001) and Interleukin 1 beta (IL1β) (Fig 4)(p<0.05). *An* anti-inflammatory cytokine, interleukin 10 (IL10), was also significantly upregulated in *H*. *pylori* infected mice (Fig 4)(p<0.0001).

### *H*. *pylori* infection altered mRNA expression profile of brain genes related to iron status, dopamine regulation, myelination, and synaptic plasticity

**Fig 5:**
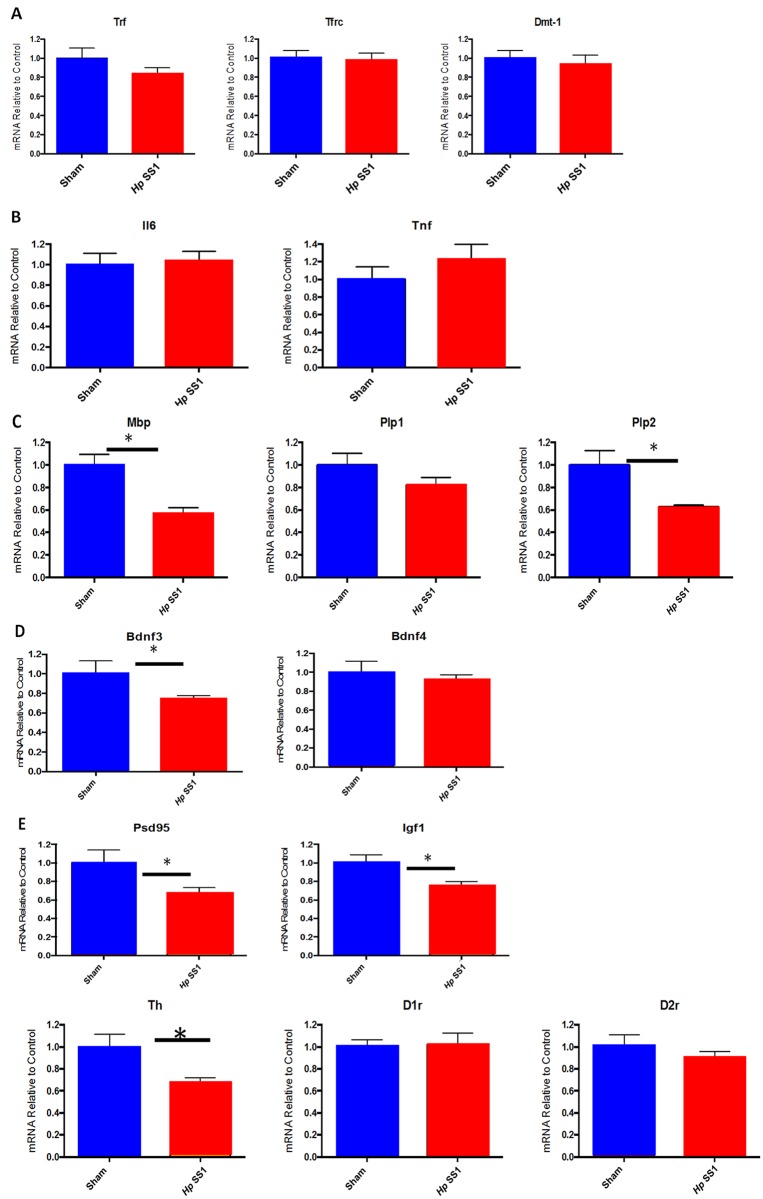
Chronic infection of male INS-GAS mice with *H*. *pylori* SS1 reduces expression of genes involved in dopamine metabolism, myelination, and synaptic plasticity without significantly altering expression of iron transport genes. (A) genes related to myelination (B) synaptic plasticity genes (C) genes involved in dopamine metabolism, (*) = p<0.05, (**) = p<0.01.

*H*. *pylori* SS1 infection did not impact expression of iron transport genes TfR and Slc11a2 in adult male mice (data not shown). Similarly, there was no difference in brain expression of the inflammatory markers IL-6 or TNFα between *H*. *pylori*-infected and control mice (S1 Fig). Expression of genes involved in myelination (myelin basic protein (*Mbp*) and proteolipid protein 2 (*Plp2*)) was significantly downregulated in *H*. *pylori*-infected mice (Fig 5A)(p = 0.001 and p = 0.02 respectively). Expression of synaptic plasticity markers (brain derived neurotrophic factor 3 (*Bdnf3*), *Psd95* (a membrane associated guanylate kinase), and insulin-like growth factor 1 (*Igf1*)) was also downregulated in *H*.*pylori*—infected mice (Fig 5B)(p = 0.09, p = 0.04, p = 0.02 respectively). *H*. *pylori* infection downregulated brain expression of tyrosine hydroxylase (*Th*), a key enzyme in dopamine synthesis (Fig 5C)(p = 0.02).

### Relative levels of synaptic plasticity-related proteins were lower in *H*. *pylori*-infected INS-GAS mice

**Fig 6:**
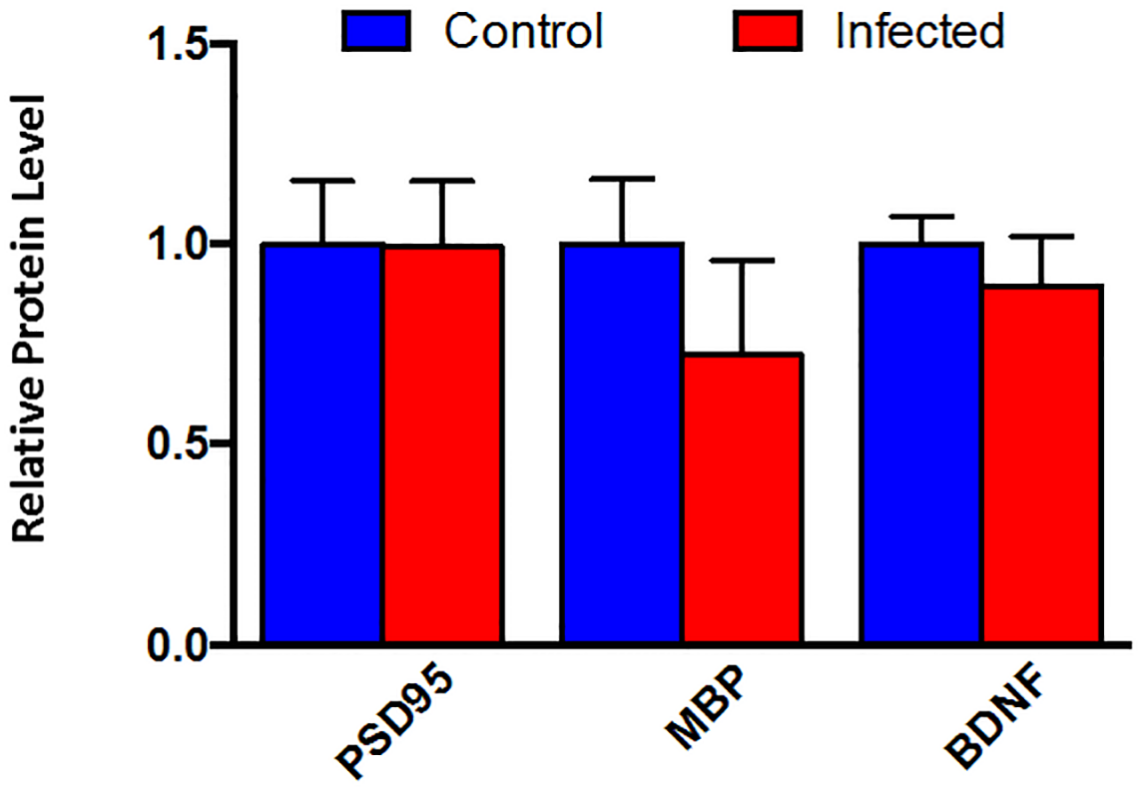
Chronic infection of male INS-GAS mice with *H*. *pylori* SS1 reduces brain protein levels of BDNF and MBP.

The relative protein levels of BDNF and MBP in brain tissue were decreased 12% and 28% respectively in INS-GAS mice infected with *H*. *pylori* when compared to sham-dosed control INS-GAS mice. (Fig 6). The protein level of PSD95 did not differ between treatment groups.

### Chronic *H*. *pylori* infection caused anemia in chronically infected INS-GAS mice

**Fig 7:**
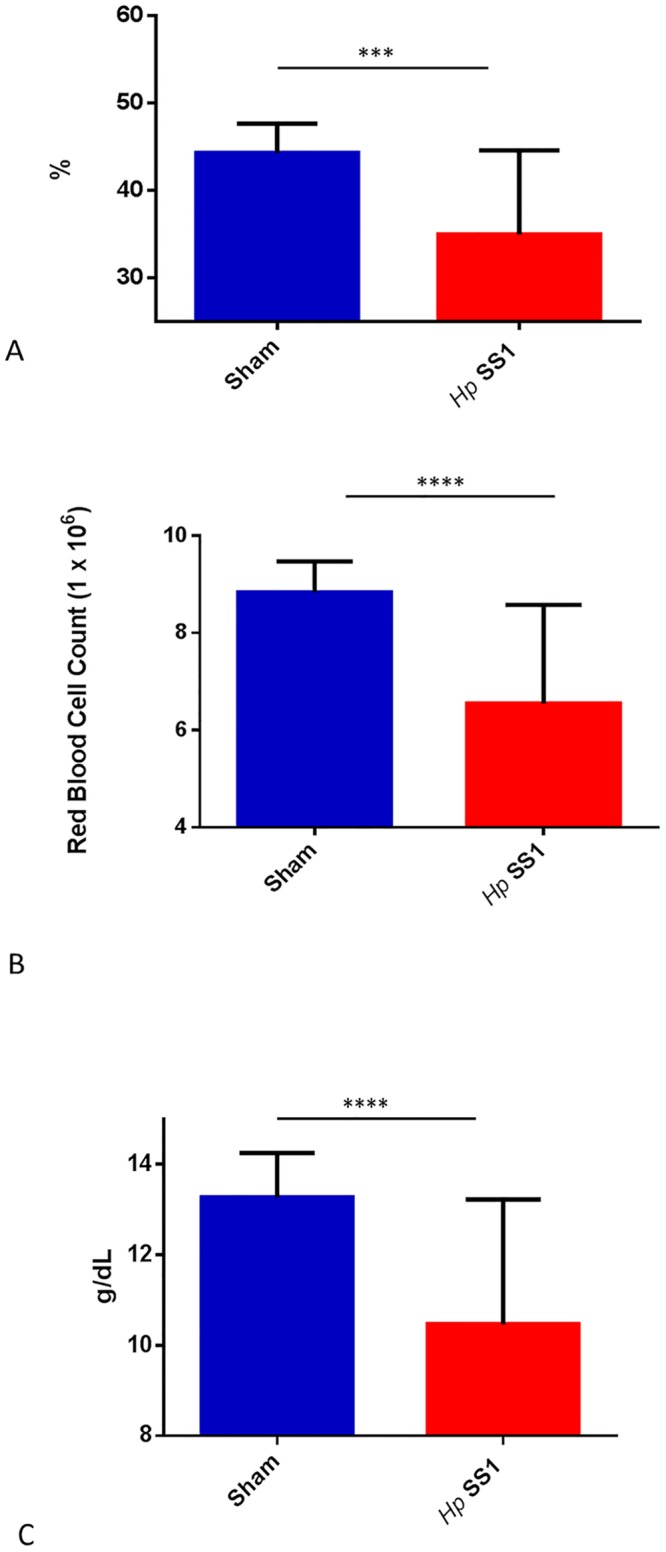
(A-C). *H*. *pylori*-infected mice have lower red blood cell indices than uninfected mice 6–7 months post infection. Mean hematocrit (A) of *H*. *pylori*-infected mice was lower than sham-dosed mice, as was red blood cell count (B) and hemoglobin concentration(C). (***) = p<0.001, (****) = p<0.0001.

Chronic infection with *H*. *pylori* caused a decrease in hematocrit, hemoglobin, and red blood cell count in male INS-GAS mice. Complete blood counts performed at study termination revealed that red blood cell number and volume were decreased in infected mice when compared to sham-dosed control mice. In both replicates of the study, hematocrit was significantly lower in mice that had been dosed with *H*. *pylori* compared to sham-dosed controls (Fig 7A)(p<0.001).

Red blood cell count was decreased in mice infected with *H*. *pylori* (Fig 7B)(p<0.0001), Hemoglobin concentration was affected in a manner similar to hematocrit and red blood cell count; hemoglobin concentration was lower in *H*. *pylori* -infected mice compared to sham-dosed controls (Fig 7C) (p<0.0001).

**Fig 8:**
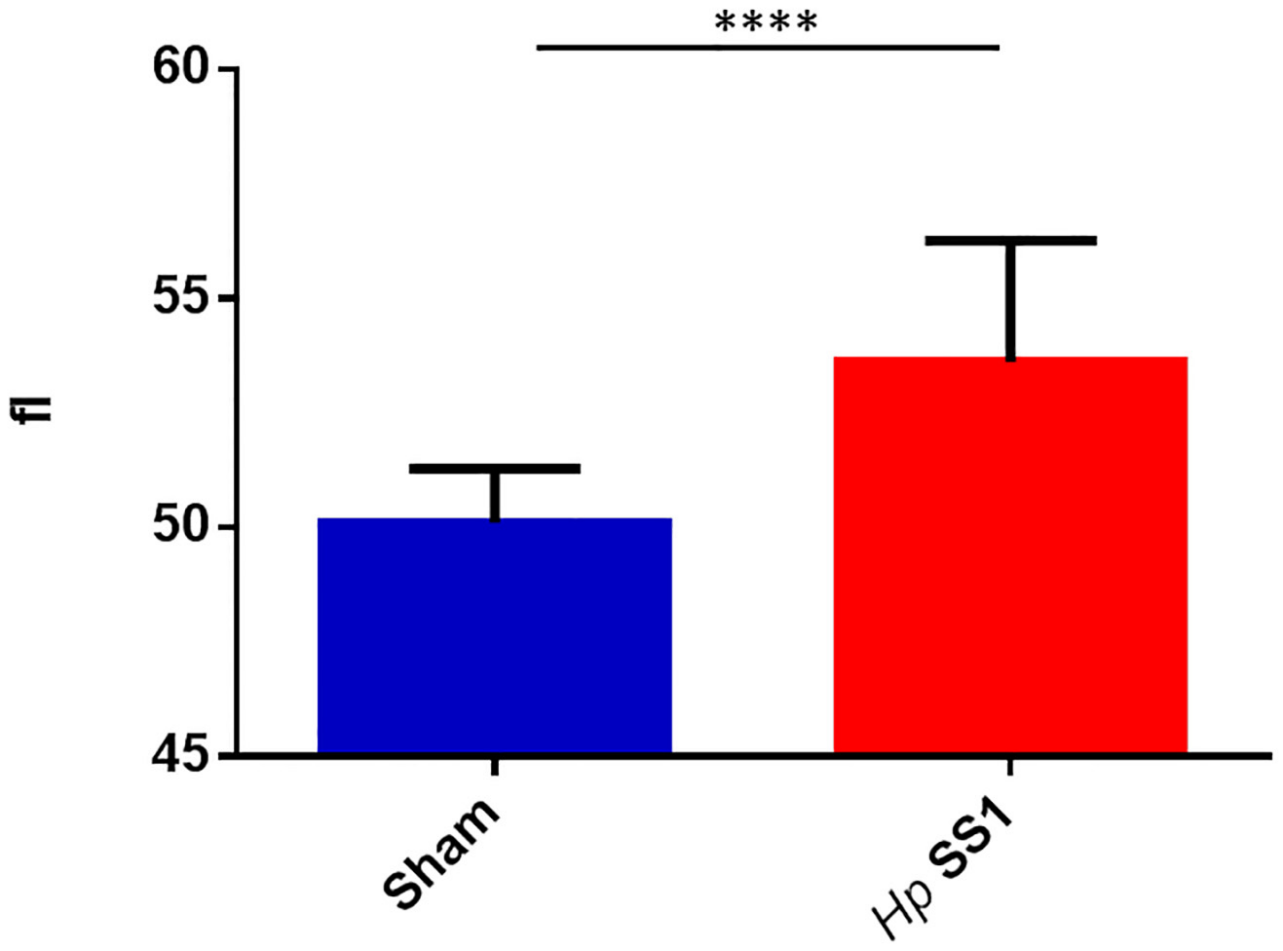
Mean cellular volume of red blood cells is higher in mice infected with *H*. *pylori* than sham-dosed mice. (****) = p<0.0001.

Mean Cellular Volume (MCV) was evaluated as a marker of red blood cell size. MCV was significantly elevated in mice infected with *H*. *pylori* compared to sham-dosed control mice (Fig 8) (p<0.0001).

### Bone marrow cytology, serum erythropoietin levels, and spleen weight reflected a regenerative response to anemia induced by *H*. *pylori*

**Fig 9:**
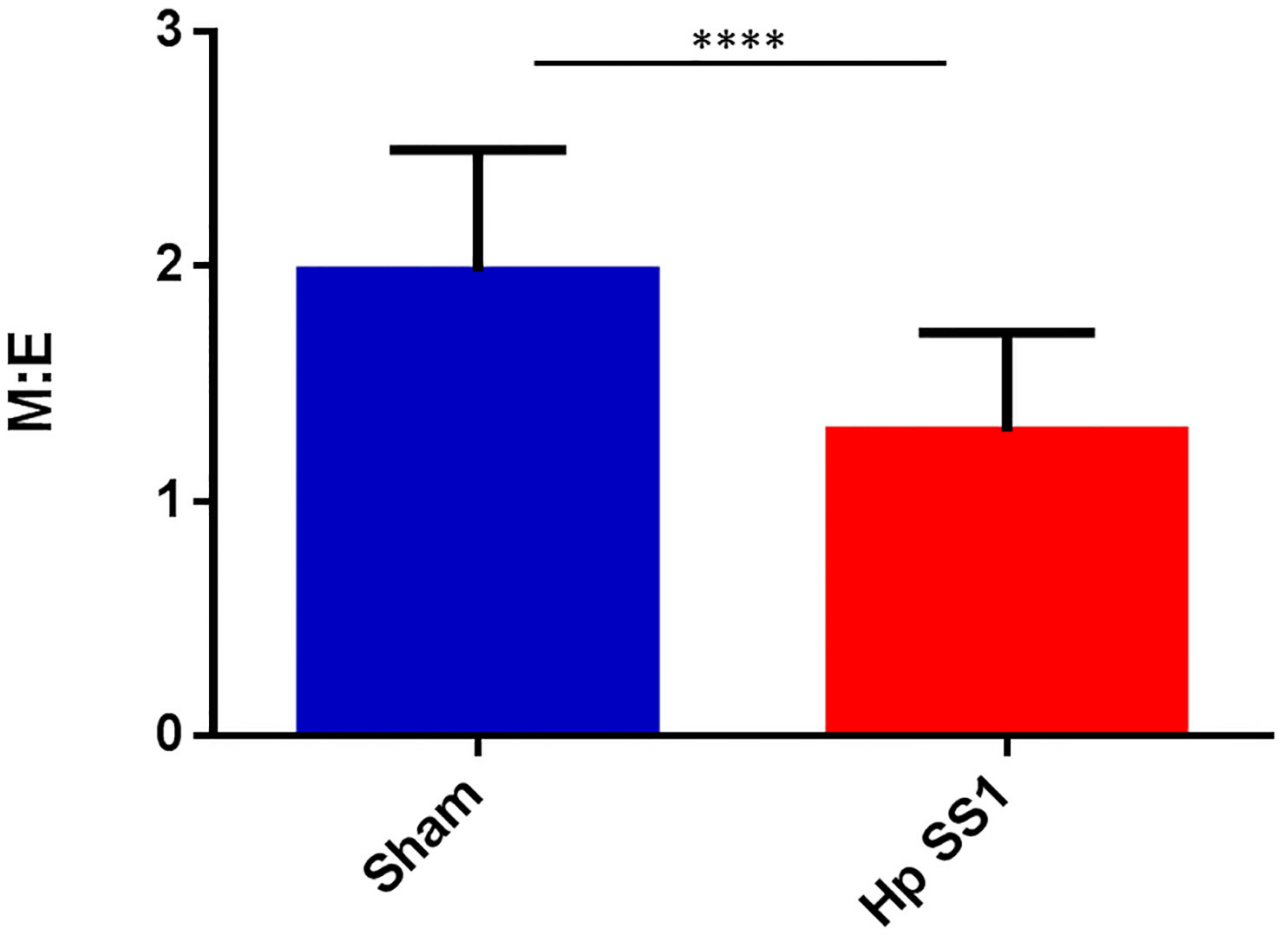
*H*. *pylori* SS1 infection decreased myeloid:erythroid precursor cell ratio on bone marrow cytology.

**Fig 10:**
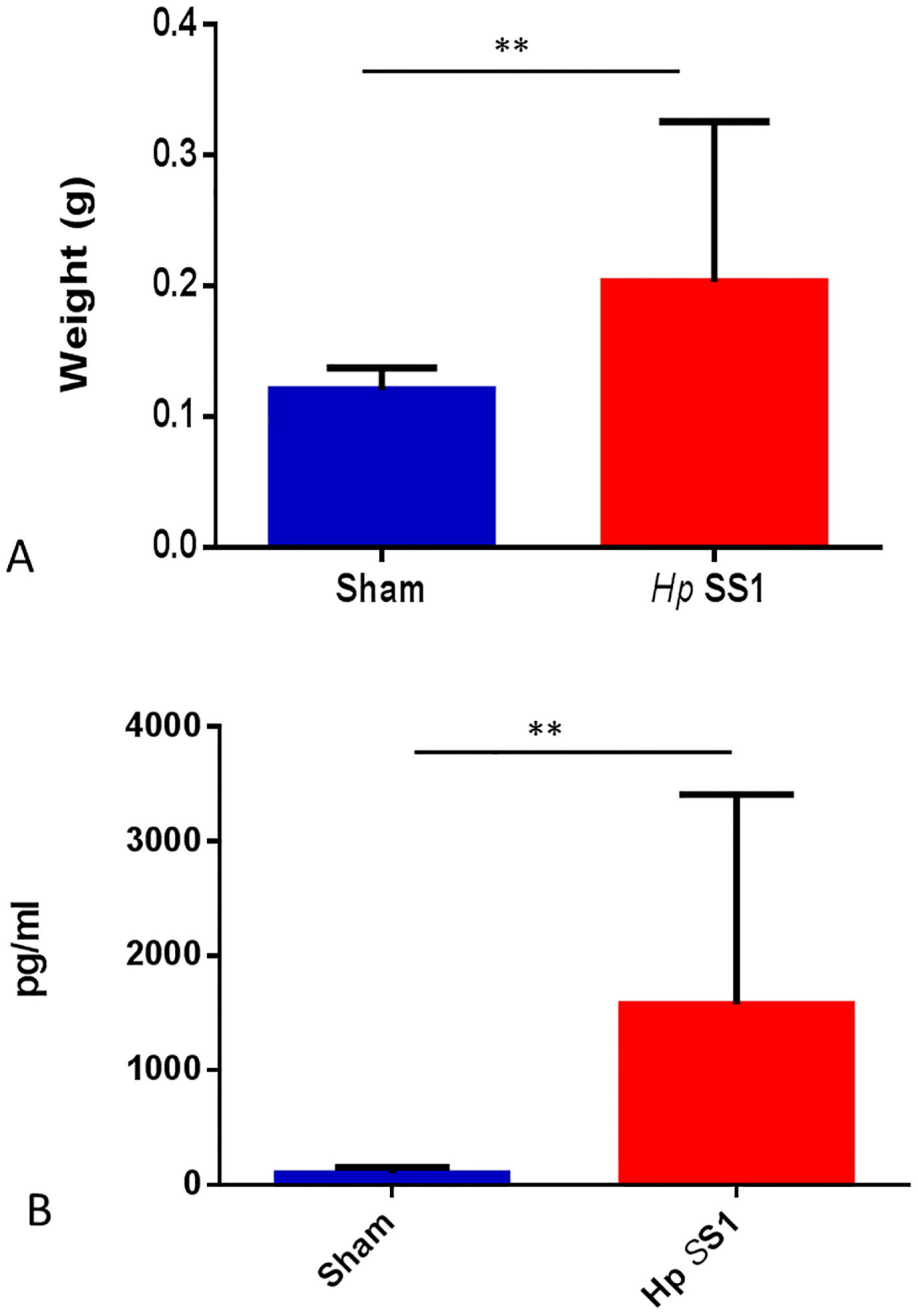
(A&B). Serum erythropoietin concentration and spleen weight are elevated in mice chronically infected with *H*. *pylori* SS1. Both mean serum erythropoietin (A) and spleen weight (B) were higher in *H*. *pylori*-infected mice than sham-dosed control mice. (**) = p<0.01.

The ratio of bone marrow myeloid to erythroid precursors was decreased in mice infected with *H*. *pylori* SS1. The relative population of myeloid to erythroid precursor cells within bone marrow was significantly decreased in mice infected with *H*. *pylori* SS1 (Fig 9: p<0.0001). *H*. *pylori* SS1 infection increased serum erythropoietin concentration in INS-GAS mice. Erythropoietin (EPO) is a hormone produced by the kidney that controls erythropoiesis. EPO concentrations were significantly increased in Study 1 mice infected with *H*. *pylori* SS1 (Fig 10A: p<0.01). Evaluation of serum EPO concentration was not performed on serum samples from study 2 mice.

Relative spleen mass and histologic evidence of extrahematopoietic erythropoiesis was higher in INS-GAS mice chronically infected with *H*. *pylori*. Spleen weight relative to body weight is used as an indicator of increased erythropoiesis/red blood cell mass within the spleen [45]. The relative mass of the spleen was significantly increased in mice infected with *H*. *pylori* SS1 when compared to sham-dosed control mice (Fig 10B) (p<0.01). Histopathologic lymphoid hyperplasia scores did not differ between infection groups, but extramedullary hematopoiesis scores were significantly elevated in *H*. *pylori*-infected INS-GAS mice compared to sham-dosed control mice (p<0.0001, data not shown).

### Chronic *H*.*pylori* infection induced significant gastric histopathological lesions

Infection with *H*. *pylori* SS1 increased severity of gastric pathology in male INS-GAS mice after 27–29 weeks of infection. Stomachs of *H*. *pylori*-infected mice in both study replicates were severely pale and thickened upon gross examination at necropsy, while stomachs of uninfected mice were mildly to moderately pale and thickened. Both infected and uninfected mice evidence of parietal cell loss; the degree of loss was subjectively more severe in infected mice (S2 Fig). *H*. *pylori*-infected mice from the first study had significantly higher mean histopathologic scores for the following categories: inflammation, epithelial defects, oxyntic atrophy, epithelial hyperplasia, pseudo-pyloric metaplasia and dysplasia (S3 Fig) (all p<0.0001) The degree of hyalinosis and mucus metaplasia observed between groups did not differ. The mean gastric histopathologic assessment index (GHAI) scores of the mice infected with *H*. *pylori* SS1 were significantly greater than GHAI scores of uninfected mice (S3 Fig)(p <0.0001).

### *H*. *pylori* established persistent colonization in INS-GAS mouse gastric tissue

Colonization levels of gastric tissue by *H*. *pylori* SS1 were evaluated at necropsy (27–29 weeks post infection). Mean relative colonization levels of mouse gastric tissue by *H*. *pylori* SS1 was 12651.0 ± 15743.9 copies of *H*. *pylori* SS1 DNA per μg of tissue.

## Discussion

The World Health Organization uses serum ferritin in the screening and diagnosis of ID and IDA, and describes this assay as “the most specific biochemical test that correlates with relative total body iron stores”[46, 47]. In this study, serum ferritin, which does not have a high turnover rate, was used as a more reliable marker of iron status than iron bound to transferrin, an iron transport protein that delivers iron from sites of absorption (duodenum) to areas with high iron demand throughout the body. Transferrin-bound iron has the highest turnover rate of any iron pool in the body, and levels are in constant flux [38]. The current finding that total serum iron did not differ between treatment groups in this study is not surprising. However, serum ferritin concentration was lower in *H*. *pylori*-infected mice than uninfected mice. Decreased serum ferritin concentrations similar to those of the current study were noted in previous mouse studies examining the impact of gastric *Helicobacter* spp infection on serum iron parameters [19, 20]. A study in gerbils that had been iron-depleted documented an increased virulence of *H*. *pylori*, as well as accelerated carcinogenesis [18]. The effects of iron deficiency were not as severe in gerbils infected with a mutant *H*. *pylori* strain that did not contain the *cag* pathogenicity island (PAI). Our study used a strain of *H*. *pylori* that did not contain a functional *cag* PAI. Although it would be interesting to evaluate the effects of a *cag+* strain of *H*. *pylori* on serum iron parameters in the INS-GAS mouse, *cag+ H*. *pylori* strains are unfortunately inefficient colonizers in mice.

Infection with *H*. *pylori* altered gene expression in gastric and hepatic tissues. The master regulator of iron metabolism is hepcidin, an antimicrobial peptide produced by and predominantly expressed in the liver. Many other tissues, such as gastric tissue, produce hepcidin as well [48]. Expression of hepcidin results in internalization and destruction of ferroportin and DMT1, two important iron transport proteins [49]. Increases in hepicidin expression result in diminished absorption of iron into enterocytes, and decreased transport to the vascular space [50]. Hepcidin has an important role as an acute phase protein in that infection and inflammation typically cause an increase in its expression. This results in decreased iron uptake, and is involved in the pathogenesis of anemia of inflammation and anemia of chronic disease. *H*. *pylori*-infected patients tend to have increased hepcidin expression as a result of chronic inflammation; however, iron deficiency results in decreased hepcidin expression, as the body compensates by obtaining more iron to increase hematopoiesis. This effect was documented in a previous study that evaluated expression of *Hamp* in *H*. *felis*-infected INS-GAS mice [19]. Infection with *H*. *pylori* resulted in significant downregulation of *Hamp* expression in gastric tissue. Liver *Hamp* expression was also found to be significantly downregulated in *H*. *pylori*-infected INS-GAS mice. These findings agree with the established response of hepcidin in the face of iron deficiency–*Hamp* expression is reduced in order to allow free flow of iron from the intestinal lumen to the enterocyte and then into the vascular space. In a similar pattern of expression, *Bmp4* was significantly downregulated in *H*. *pylori*-infected animals. This finding also matches the *Bmp4* expression pattern noted in INS-GAS mice infected with *H*. *felis* [19]. Given that *Bmp4* expression is an upstream regulator of *Hamp*, it is expected to rise and fall in a manner similar to *Hamp* expression.

*H*. *pylori* infection also disrupted brain gene expression in INS-GAS mice. Appropriate brain iron homeostasis is essential to both energy metabolism and neurotransmitter homeostasis; humans that experience ID, especially during periods of growth (high demand), are prone to development of a host of neurological sequelae including cognitive, emotional, and behavioral problems [51, 52]. Understanding the myriad effects of ID on the brain is critical, as individuals that experience ID during crucial periods of brain development and high iron demand experience long-term negative effects years after iron repletion. It is not surprising that no differences were noted in expression of either transferrin receptor (*TfR*) or divalent metal ion transporter 1 (*Slc11a2*) in *H*. *pylori*-infected adult mice. By adulthood, there is little need to transport iron from the vascular space into brain tissue, as the growth-intensive stages of life, which require abundant iron, have been completed. The lack of change in expression of inflammatory markers in infected mice is reflective of a non-inflammatory process affecting the brain. However, other domains of gene expression were altered in *H*. *pylori*-infected adult mice. For example, expression of tyrosine hydroxylase, an important enzyme in dopamine synthesis, was significantly downregulated in *H*. *pylori*-infected mice compared to uninfected mice. Previous studies have established that iron deficiency has detrimental short and long-term effects on dopamine metabolism [53]. Dopamine is critical in learning and memory processes, and disruption of dopamine metabolism may result in cognitive deficiencies. Similarly, expression of genes related to both myelination and synaptic plasticity was downregulated in *H*. *pylori*-infected mice. Homeostatic synaptic plasticity is a critical process that allows neurons to adjust their level of excitability [54]. The animals in this study were maintained on an iron-replete diet, and therefore it is unlikely they experienced significant brain iron deficiency until persistent *H*. *pylori* infection was established. It is interesting that alterations in brain expression of genes related to dopamine metabolism, myelination, and synaptic plasticity were still observed in *H*. *pylori*-infected adult mice. While the cognitive effects of ID are indisputably more severe when ID is experienced during neonatal or juvenile periods of growth and development, the results of this study reinforce the concept that ID can impact the adult brain as well.

Although previous studies have examined the effect of gastric *Helicobacter spp*. infection on serum iron biomarkers [19, 20], this study is the first to evaluate the effect of gastric *H*. *pylori* infection on hematological parameters in the INS-GAS mouse model. Although wild type FVB/N mice were not included in our study, the WT FVB/N results of the Thomson et al study can be used as a WT reference for many of the iron-related serum and gene expressionparameters examined in our experiment. In the current study, INS-GAS mice infected with *H*. *pylori* had significantly lower hematocrit, hemoglobin concentration, and red blood cell counts than uninfected control mice. The mean hematocrit, hemoglobin concentration, and red blood cell counts of uninfected mice from this study are equivalent to previously published normal hematologic values of FVB male mice [55]. Based on red blood cell indices, the *H*. *pylori*-infected mice in this study experienced a mild to moderate decrease in red blood cell mass, indicating red blood cell indices consistent with mild to moderate anemia.

Hematologic parameters evaluated in this study, such as MCV, suggest that the mild to moderate anemia noted in the infected mice is a regenerative anemia rather than the microcytic, non-regenerative anemia typically documented in most cases of iron deficiency anemia. Hemorrhage that is acute or short in duration results in an increased release of immature red blood cells (reticulocytes) into the vascular compartment. Since reticulocytes are larger in size than mature red blood cells, this causes an increase in mean cellular volume (MCV) [56]. The findings of our study are therefore consistent with acute, short duration hemorrhage, visually confirmed in one *H*. *pylori*-infected mouse at necropsy. Mice infected with *H*. *pylori* SS1 were noted to have a higher reticulocyte percentage (data not shown) and MCV in the face of decreased hematocrit, red blood cell count, and hemoglobin than uninfected mice. These findings have been reported previously in rodent studies of hemorrhage and phlebotomy [57]. Humans, mice, and other animals with gastrointestinal neoplasia are prone to blood loss from preneoplastic or neoplastic lesions that erode through GI epithelium and expose the vascular space [58]. This blood loss begins acutely and becomes chronic over time. Chronic blood loss causes a depletion of iron, and results in the development of microcytic, hypochromic iron deficiency anemia, as the initial increased erythropoietic response is eventually unable to keep up with the body’s demand for additional red blood cells to compensate for those lost to chronic hemorrhage [59]. Thus, a process that began with acute blood loss and a macrocytic, normochromic anemia will eventually transition to a chronic blood loss represented by a microcytic, hypochromic anemia of iron deficiency. Patients with gastrointestinal diseases, particularly those that involve bleeding, have a high prevalence of iron deficiency anemia [60].

Serum erythropoietin concentration was also elevated in *H*. *pylori—*infected mice compared to sham-dosed control mice in Study 1. Erythropoietin (EPO), a hormone produced by the kidney, acts as a regulator of erythropoiesis via promotion of differentiation of erythroid precursors [61]. Increased levels of EPO result in an increase in red blood cell production and hemoglobin levels. EPO levels are low in the absence of anemia, but increase dramatically in cases of hypoxic stress due to decreased blood cell mass. Other studies have shown increased EPO in mice with anemia [62]. To our knowledge, the current study represents the first evaluation of serum EPO concentration in *H*. *pylori*-infected mice [45]. In mice, the spleen is a highly erythropoietic organ, particularly in the presence of anemia. The increased splenic mass noted in the *H*. *pylori*-infected animals in the current study is consistent with previous studies that have reported splenomegaly after induction of anemia and iron deficiency in various mouse strains [63]. Histopathologic analysis revealed the presence of significantly increased extramedullary hematopoiesis in spleens from *H*. *pylori*-infected mice, a finding consistent with a regenerative response to anemia. The bone marrow response to anemia was also regenerative in this study, as evidenced by an increased ratio of myeloid to erythroid precursors present. To document the development of iron deficiency anemia, the period of *H*. *pylori* infection prior to necropsy should be extended to longer than 6.5–7 months.

In humans, *H*. *pylori* infection causes persistent chronic gastritis, resulting in parietal cell loss and a mild (1.5-2-fold elevation) hypergastrinemia. In susceptible individuals, this chronic gastritis may progress to gastric cancer [64]. Hypergastrinemic INS-GAS mice develop gastric cancer at 6–7 months post-infection with *H*. *pylori*, through a disease process that mimics *H*. *pylori–*related gastric cancer in humans [35, 65]. In this study, while our aim was to define the hematologic and iron-related gene expression outcomes of chronic *H*. *pylori* infection in INS-GAS mice, it is also important to consider the effect of gastrin both on iron homeostasis and the development of gastric dysplasia. Circulating gastrin concentration has been shown to alter iron homeostasis in both humans and juvenile mice [23]. This is due to the effect of gastrin on intestinal absorption of dietary iron, which is a crucial regulatory point of iron homeostasis. While marked differences were noted in serum iron parameters in juvenile transgenic mice with abnormal serum gastrin, these differences normalized by adulthood. Hypergastrinemic humans have also been shown to have high transferrin saturation that correlates with circulating gastrin concentrations [23]. Though elevated levels of gastrin certainly may have an effect on iron homeostasis in the INS-GAS mouse, it is unlikely to have affected the outcome of our study, as we used mice that were well into adulthood. Therefore, we do not expect that the disturbances in iron homeostasis in *H*. *pylori*-infected INS-GAS mice documented in this study were caused by elevated serum gastrin.

The histopathologic changes noted in the current study are consistent with previous studies using *H*. *pylori*-infected INS-GAS mice [35, 36]. Our study and previous studies have shown that infection of male INS-GAS/FVB mice with *H*. *pylori* results in the development of glandular atrophy, intestinal metaplasia, hyperplasia, dysplasia, and cancer [44]. Glandular atrophy causes depletion of parietal cells, which leads to hypochlorhydria, causing an increase in gastric pH [19]. Severe loss of parietal cells in *H*. *pylori*-infected INS-GAS mice is likely responsible for the depletion of iron stores documented in the current study, as a low gastric pH is required for the reduction of iron from the non-absorbable ferric state to the absorbable ferrous state. A common sequela of male, *H*. *pylori*-infected INS-GAS mice is a propensity for development of gastric adenocarcinoma, accompanied by epithelial erosions, and intermittent, chronic blood loss. In humans, many individuals with a history of gastric ulceration ultimately develop gastric cancer as well [66]. While fecal occult blood status was not evaluated in this study, gastric hemorrhage was grossly evident in the stomach of a *H*. *pylori*-infected mouse during necropsy, as was significant epithelial erosions in other infected mice; these gastric lesions and blood in the gastric lumen have been noted in our previous INS-GAS studies [67, 68]. This finding suggests that *H*. *pylori*-infected mice experienced episodic gastric hemorrhage.

In summary, INS-GAS mice infected with *H*. *pylori* SS1 develop a mild to moderate, regenerative anemia after being infected for 6.5–7 months. The consistency of results in the two studies strengthens our conclusion that male INS-GAS mice infected with *H*. *pylori* can serve as an animal model for iron deficiency and anemia due to *H*.*pylori* infection. Behavioral assessment of the effects of chronic *H*. *pylori* infection and subsequent anemia and iron deficiency in mice will provide to further characterization of the cognitive impact of iron deficiency in *H*. *pylori*-infected hosts.

## Supporting Information

S1 Fig Brain expression of inflammatory cytokines was not affected by *H*. *pylori* infection. A) IL-6 B) TNFa.
(TIF)

S2 Fig Gastric histopathologic lesions were more severe in mice infected with *H*. *pylori* than uninfected control mice. Magnification: 40x. Bar: 400μM.
(TIF)

S3 Fig *H*. *pylori*-infected mice had significantly higher mean histopathologic scores than uninfected control mice. (**** = p<0.0001).
(TIF)
