# Supplementary figures and images for: Helicobacter pylori Infection Induces Anemia, Depletes Serum Iron Storage, and Alters Local Iron-Related and Adult Brain Gene Expression in Male INS-GAS Mice
Source: PLoS One. 2015 Nov 17;10(11):e0142630. doi: 10.1371/journal.pone.0142630 (PMC4648568; doi:10.1371/journal.pone.0142630)

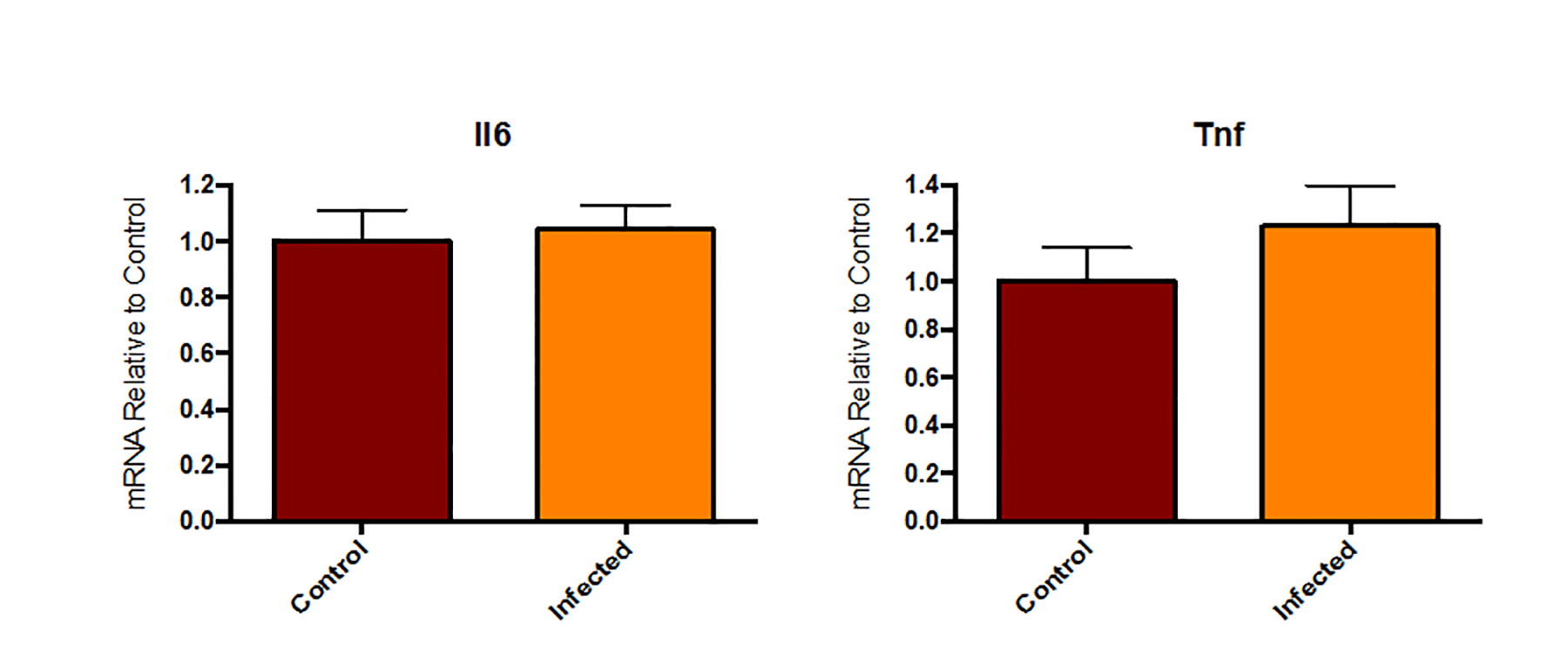

Supplement: S1 Fig — A) IL-6 B) TNFa. (TIF) [file pone.0142630.s001.tif]

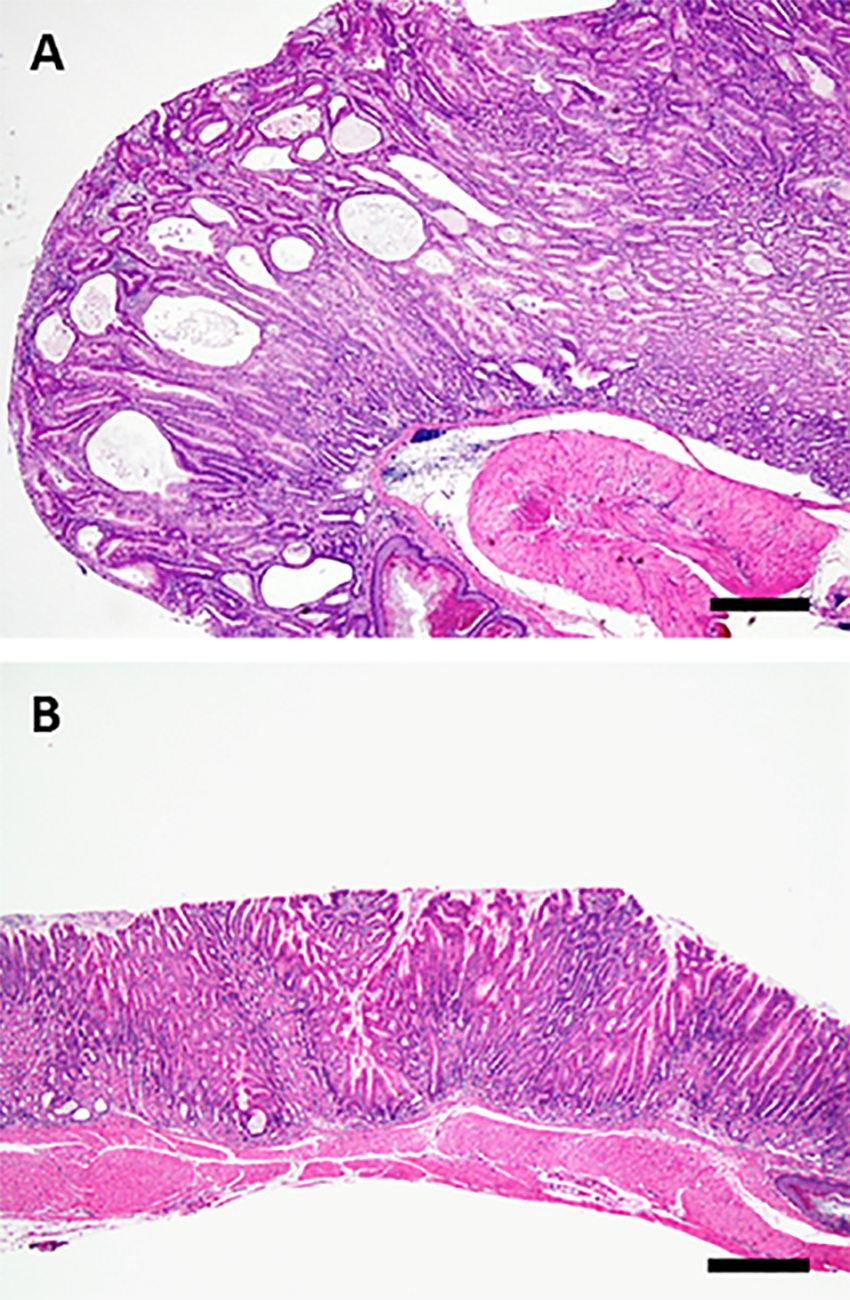

Supplement: S2 Fig — Magnification: 40x. Bar: 400μM. (TIF) [file pone.0142630.s002.tif]

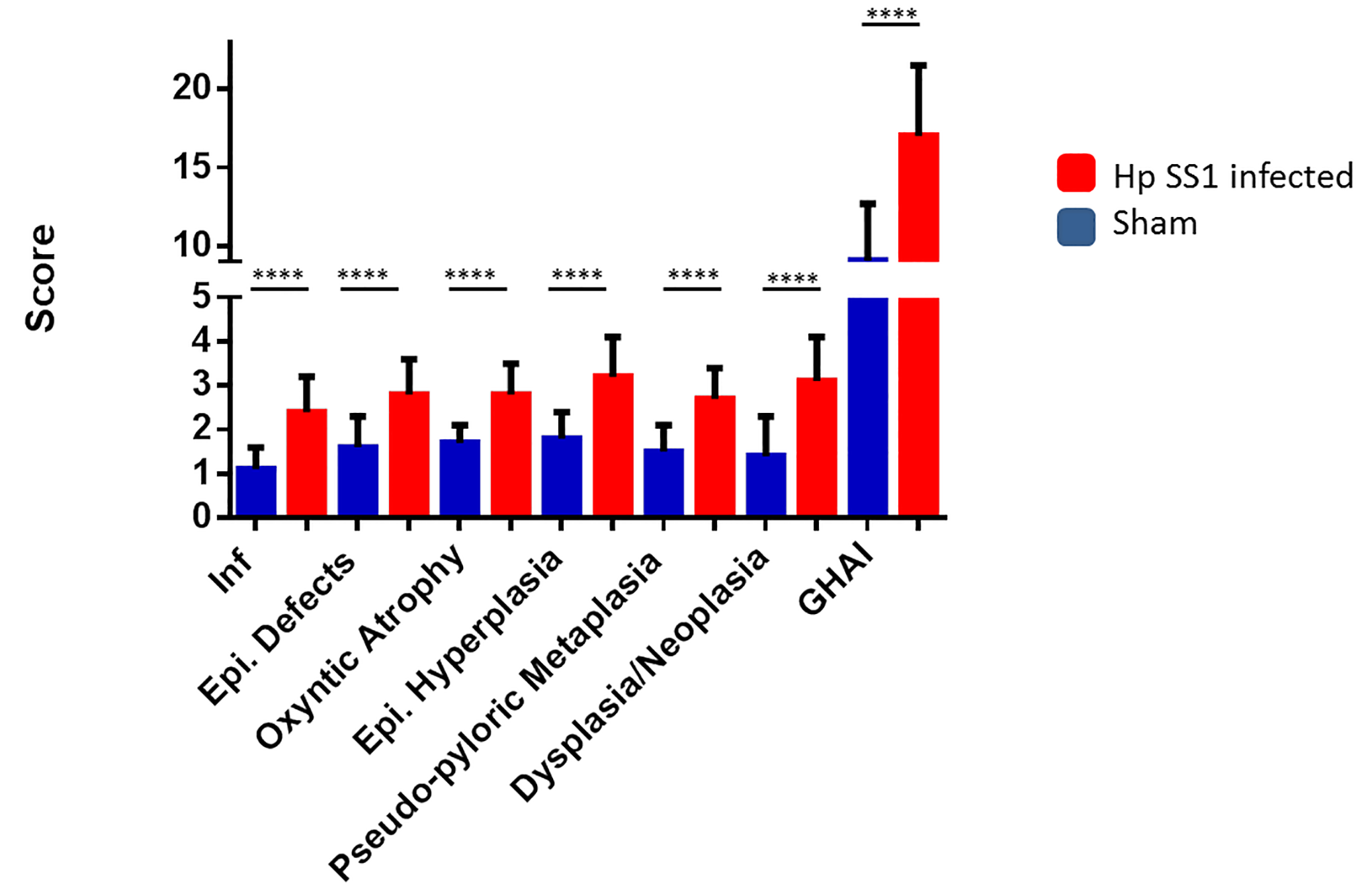

Supplement: S3 Fig — (**** = p<0.0001). (TIF) [file pone.0142630.s003.tif]
